# Additional oncological benefit of photodynamic diagnosis with blue light cystoscopy in transurethral resection for primary non‐muscle‐invasive bladder cancer: A comparative study from experienced institutes

**DOI:** 10.1002/bco2.215

**Published:** 2023-01-13

**Authors:** Makito Miyake, Nobutaka Nishimura, Tomonori Nakahama, Koshiro Nishimoto, Masafumi Oyama, Yuto Matsushita, Hideaki Miyake, Hideo Fukuhara, Keiji Inoue, Keita Kobayashi, Hiroaki Matsumoto, Hideyasu Matsuyama, Tomomi Fujii, Yoshihiko Hirao, Kiyohide Fujimoto

**Affiliations:** ^1^ Department of Urology Nara Medical University Kashihara Nara Japan; ^2^ Department of Uro‐Oncology Saitama Medical University International Medical Center Hidaka Saitama Japan; ^3^ Department of Urology Hamamatsu University School of Medicine Hamamatsu Shizuoka Japan; ^4^ Department of Urology Kochi Medical School Nankoku Kochi Japan; ^5^ Department of Urology, Graduate School of Medicine Yamaguchi University Ube Yamaguchi Japan; ^6^ Department of Diagnostic Pathology Nara Medical University Kashihara Nara Japan; ^7^ Department of Urology Osaka Gyoumeikan Hospital Konohana‐ku Osaka Japan

**Keywords:** aminolevulinic acid, disease progression, local, neoplasm recurrence, optical imaging, photodynamic diagnosis, urinary bladder neoplasms

## Abstract

**Objectives:**

The objective of this work is to evaluate the additional oncological benefit of photodynamic diagnosis (PDD) using blue‐light cystoscopy in transurethral resection (TURBT) for primary non‐muscle‐invasive bladder cancer (NMIBC) based on the International Bladder Cancer Group (IBCG)‐defined progression and the subsequent pathological pathways.

**Patients and Methods:**

We reviewed 1578 consecutive primary NMIBC patients undergoing white‐light TURBT (WL‐TURBT) or PDD‐TURBT during 2006–2020. One‐to‐one propensity score‐matching was performed using multivariable logistic regression to obtain balanced groups. IBCG‐defined progression of NMIBC included stage‐up and grade‐up as well as conventional definitions such as the development of muscle‐invasive BC or metastatic disease. Nine oncological endpoints were evaluated. Sankey diagrams were generated to visualize follow‐up pathological pathways after the initial TURBT.

**Results:**

Comparison of event‐free survival between the matched groups revealed that PDD use decreased the bladder cancer recurrence risk and IBCG‐defined progression risk, whereas no significant difference was noted in conventionally defined progression. This was attributable to a reduced risk of stage‐up, from Ta to T1, and grade‐up. Sankey diagrams of the matched groups showed that patients with primary Ta low‐grade tumour and first‐recurrence Ta low‐grade tumour did not have bladder recurrence or progression, while some of those in the WL‐TURBT group developed recurrence after treatment.

**Conclusions:**

The multiple survival analysis demonstrated that the risk of IBCG‐defined progression was significantly decreased by PDD use in NMIBC patients. Sankey diagrams revealed possible differences in pathological pathways after the initial TURBT between the two groups, demonstrating that repeated recurrence could be prevented by PDD use.

## 1 INTRODUCTION

Current guidelines recommend the use of photodynamic diagnosis (PDD) with blue‐light cystoscopy in transurethral resection of bladder tumours (TURBTs) and guided bladder biopsy for the treatment and diagnosis of non‐muscle‐invasive bladder cancer (NMIBC).^1^, ^2^, ^3^ Systematic reviews and meta‐analyses of PDD‐TURBT have shown that it achieves a higher detection rate of malignant tumours, particularly small lesions and carcinoma in situ (CIS), than does the conventional white‐light (WL) procedure, leading to a reduction in bladder recurrence rates.^4^, ^5^, ^6^, ^7^ However, progression and mortality rates were similar.

Progression, that is, the worsening of a disease, is another key clinical outcome of NMIBC. Most previous studies have defined progression as the development of muscle‐invasive BC (MIBC), lymph node metastasis (N+), or distant metastasis (M+). For a more accurate comparison of treatment options, progression in NMIBC additionally needs to include ‘worrisome’ bladder recurrence, as this could change further intervention and follow‐up intensity. In 2014, the International Bladder Cancer Group (IBCG) proposed a new definition of progression in NMIBC, which included tumour stage‐up and grade‐up.^8^ However, real‐world evidence regarding the benefit of using PDD use based on the revised definition is scarce.

PDD is performed using violet‐blue light after intravesical instillation of 5‐aminolaevulinic acid (ALA) or hexaminolevulinic acid (HAL) in US and European countries, whereas oral ALA was approved as an intraoperative diagnostic drug in Japan based on clinical trial evidence.^9^, ^10^ Our collaborative group has accumulated experience with ALA‐mediated PDD‐TURBT since 2004. Here, large‐scale data from these experienced institutes were analysed to evaluate the clinical impact of PDD‐TURBT on bladder recurrence risk, conventionally defined progression (c‐progression), progression based on IBCG criteria (IBCG‐progression), development of metastatic disease, and overall mortality in primary NMIBC patients. Moreover, as real‐world data regarding pathological pathways from primary NMIBC to subsequent progression are limited, we generated Sankey diagrams and swimmer plots to investigate possible differences in the pathological pathways between the WL‐TURBT and PDD‐TURBT groups, to improve understanding of how patients with primary NMIBC developed disease progression.

## 2 METHODS

### 2.1 Patients and data collection

This retrospective multicentre study was approved by each participating institute's ethics committee (reference ID: 2949). The study was conducted in compliance with the study's protocol and in accordance with the provisions of the Declaration of Helsinki (2013). Informed consent was obtained from the participants or deceased patients' families through posters and/or websites using the opt‐out method.

We reviewed 1578 consecutive patients with primary NMIBC who underwent WL‐TURBT or PDD‐TURBT, from February 2006 to December 2020, at Nara Medical University Hospital, Saitama Medical University International Medical Center, Hamamatsu University Hospital, Kochi Medical School Hospital, and Yamaguchi University Hospital. The recorded clinicopathological characteristics of the patients included age, sex, performance status (ECOG‐PS), smoking history, tumour size, multiplicity, T category, tumour grade, lymphovascular invasion, CIS, prostate‐involving CIS, variant histology, intravesical treatment, and follow‐up data. The patients undergoing immediate cystectomy for primary high‐risk NMIBC were excluded from studied cohort.

### 2.2 Surgery, postoperative therapy, and follow‐up

The preoperative process and surgical procedure for the administration of ALA, anaesthesia, resection device, and imaging devices for WL‐TURBT and PDD‐TURBT have been described previously.^9^, ^10^, ^11^ Approximately 3 h (range, 2–4 h) presurgery, patients orally received ALA hydrochloride in water (SBI Pharmaceuticals Co., Ltd., Tokyo, Japan), at a dose of 20 mg/kg. Immediate post‐TUR intravesical chemotherapy with anthracyclines and therapeutic or adjuvant intravesical bacillus Calmette–Guérin (BCG) were administered at the physician's discretion. Patients were essentially followed‐up with routine cystoscopy combined with urine cytology and imaging at regular intervals, according to the contemporary clinical practice guidelines.

### 2.3 Assessment of multiple oncological outcomes

Bladder recurrence was defined as recurrent intravesical tumours of pathologically proven urothelial carcinoma. C‐progression included development of MIBC, N+, or M+. IBCG‐progression included any one of (i) an increase in T stage from Ta to Tis or T1, or Tis to T1, (ii) an increase in tumour grade from low‐grade (LG) to high‐grade (HG, including CIS), and (iii) c‐progression.^8^, ^12^ Because an increase in tumour grade from LG to HG (including CIS) was defined ‘grade‐up’ in the IBCG report published in 2014,^8^ an increase in tumour grade from WHO1973 G1 to G2 or from G2 to G3 were not considered ‘grade‐up’ in this study.

### 2.4 Statistical analysis

Baseline clinicopathological characteristics and progression patterns were compared using the Mann–Whitney *U*, chi‐square, or Fisher's exact tests, as appropriate. Baseline characteristics were matched by calculating the propensity score for each patient using multivariable logistic regression, using potential covariates, as previously described.^13^ Propensity score matching (PSM) was performed using EZR version 1.55.^14^ One‐to‐one matching with a calliper‐width of 0.2 was applied to maintain a large sample size while balancing covariates between the WL‐TURBT and PDD‐TURBT groups. Standardized mean difference was used to examine the balance of covariate distribution between the groups after PSM.

Nine oncological endpoints were evaluated: Bladder recurrence‐free survival (BRFS), c‐progression‐free survival (c‐PFS), IBCG‐progression‐free survival (IBCG‐PFS), stage‐up‐free survival, grade‐up‐free survival, HG tumour bladder recurrence‐free survival (HG‐BRFS), MIBC‐free survival, metastasis‐free survival, and overall survival (OS). Survival was calculated from the date of initial TURBT. Survival rates were estimated using the Kaplan–Meier method and were compared using the log‐rank test. Statistical analyses and graphing were performed using GraphPad Prism version 9.4.1 (GraphPad Software, San Diego, CA, USA). All reported *P* values were two‐sided, and statistical significance was set at *P* < 0.05.

Both bladder recurrence and IBCG‐progression can occur repeatedly and metachronously in the same patient: For example, primary TaLG to recurrent TaHG, and then, recurrent T1HG. Thus, we utilized the person‐time method to compare the cumulative incidence risk of bladder recurrence and IBCG‐progression after initial TURBT between the two groups.^11^ The incidence rates were compared by incidence rate ratio (Clopper–Pearson confidence interval [CI]) and the exact *P* value conditioned on the total number of cases, which followed a Poisson distribution.^15^, ^16^, ^17^

Sankey diagrams were used to provide a visual depiction of the pathological pathway after primary NMIBC. For simplicity, only patients who experienced recurrence or progression at least once were included in these diagrams. Data preparation and graphing were performed using R version 4.2.0 (packages: tidyverse, survival; https://www.r-project.org/).^18^, ^19^ As Sankey diagrams do not reflect treatment duration and sequence, or treatment switching timing, we generated swimmer plots to show treatment sequences, pathways to progression, survival, and death during follow‐up only for patients who had IBCG‐defined progression.

## 3 RESULTS

**FIGURE 1:**
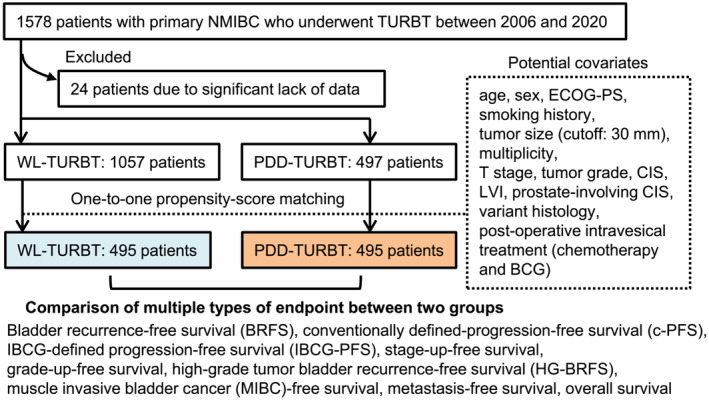
Study design and flow chart of patient selection and matching. The patient selection and propensity‐score matching process for creation of the datasets are shown. Multiple types of oncological endpoints were evaluated in this study. NMIBC, non‐muscle‐invasive bladder cancer; TURBT, transurethral resection of bladder tumour; WL, conventional white‐light; PDD, photodynamic diagnosis‐assisted; ECOG‐PS, Eastern Cooperative Oncology Group Performance Status; CIS, carcinoma in situ; LVI, lymphovascular invasion; BCG, bacillus Calmette‐Guerin

Figure 1 shows the flowchart of the patient selection and PSM process. The 1554 patients with sufficient baseline data and follow‐up information were divided into the WL‐TURBT (*n* = 1057) and PDD‐TURBT (*n* = 497) groups. Sex, tumour size, and variant histology differed statistically significantly between the unmatched groups (Table S1). One‐to‐one PSM (complete case analysis) extracted 495 patients in each group with a closely balanced distribution of baseline covariates. The median follow‐up period was 45.0 months (interquartile range, 16–68).

**FIGURE 2:**
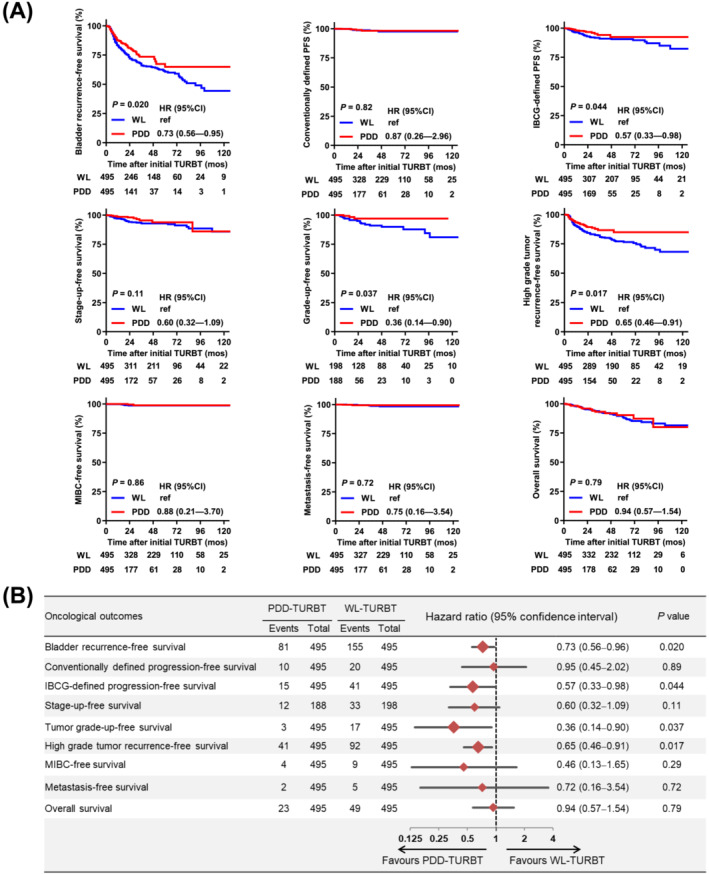
Comparison of oncological outcomes between the propensity score‐matched WL‐TURBT and PDD‐TURBT groups. (A) Survival curves for nine oncological outcomes were generated from the date of initial TURBT (diagnosis of bladder cancer). (B) Forest plots comparing oncological outcomes between the WL‐TURBT and PDD‐TURBT groups. Survival rates were estimated using the Kaplan–Meier method and hazard ratios (HRs) with 95% confidence intervals (CIs) were calculated using the log‐rank test. Grade‐up‐free survival was evaluated only in 386 patients with primary low‐grade NMIBC. MIBC, muscle‐invasive bladder cancer; TURBT, transurethral resection of bladder tumour; WL, conventional white‐light; PDD, photodynamic diagnosis‐assisted

Survival curve comparison of the nine endpoints between the two PSM‐adjusted groups revealed that PDD use improved BRFS (*P* = 0.02), IBCG‐PFS (*P* = 0.044), grade‐up‐free survival (*P* = 0.037), and HG‐BRFS (*P* = 0.017), whereas no difference was noted in c‐PFS (*P* = 0.82), MIBC‐free survival (*P* = 0.86), metastasis‐free survival (*P* = 0072), or OS (*P* = 0.79) (Figure 2). Figure S1 compares the survival curves for the nine endpoints between the unmatched groups.

Cumulative incidence rates of bladder recurrence and each IBCG‐progression pattern at the 1‐, 2‐, 3‐, 5‐, and 10‐year follow‐up are compared between the adjusted groups in Table 1. At the 2‐year follow‐up, the incidence rate ratio of bladder recurrence and IBCG‐defined progression was significantly lower in the PDD‐TURBT than in the WL‐TURBT group (*P* = 0.047 and *P* = 0.007, respectively). Similar findings in were observed in the long‐term follow‐up analyses of IBCG‐defined progression (3‐, 5‐, and 10‐year follow‐up). This was particularly pronounced in patients progressing from Ta to T1 (Table 1) and those progressing from LG to HG tumours (Figure 2). Thirty‐four patients (6.9%) and 10 patients (2.0%) in the WL‐TURBT and PDD‐TURBT groups, respectively, who were classified as non‐progression bladder recurrence based on the conventionally used definition, experienced clinically significant ‘worrisome’ bladder recurrence according to the IBCG definition.

**TABLE 1:** Comparison of cumulative incidence of bladder recurrence and progression at 1, 2, 3, 5, and 10 years after initial TURBT in patients with primary NMIBC

Patterns of progression	1‐year follow‐up				2‐year follow‐up				3‐year follow‐up				5‐year follow‐up				10‐year follow‐up			
	WL	PDD	Incidence rate ratio (Clopper–Pearson CI)	Exact *P* value	WL	PDD	Incidence rate ratio (Clopper–Pearson CI)	Exact *P* value	WL	PDD	Incidence rate ratio (Clopper–Pearson CI)	Exact *P* value	WL	PDD	Incidence rate ratio (Clopper–Pearson CI)	Exact *P* value	WL	PDD	Incidence rate ratio (Clopper–Pearson CI)	Exact *P* value
Bladder recurrence	81	59	0.77 (0.54–1.09)	0.15	143	92	0.76 (0.58–0.99)	0.047	175	103	0.81 (0.63–1.04)	0.094	207	113	0.90 (0.71–1.14)	0.42	222	114	0.95 (0.75–1.20)	0.66
Conventionally defined PFS	1	1	1.06 (0.01–83.1)	0.74	6	3	0.59 (0.10–2.8)	0.34	7	5	0.98 (0.25–3.6)	0.61	7	5	1.18 (0.30–4.33)	0.50	7	5	1.32 (0.33–4.84)	0.42
IBCG‐defined PFS	17	8	0.50 (0.19–1.2)	0.07	33	12	0.43 (0.20–0.86)	0.007	42	15	0.49 (0.25–0.90)	0.01	43	16	0.62 (0.32–1.12)	0.06	50	16	0.59 (0.31–1.06)	0.04
Stage‐up																				
Ta to Tis	3	2	0.71 (0.06–6.2)	0.53	6	2	0.39 (0.04–2.2)	0.21	10	2	0.27 (0.03–1.29)	0.06	10	3	0.50 (0.09–1.93)	0.22	13	3	0.43 (0.08–1.55)	0.13
Ta to T1	10	3	0.32 (0.06–1.2)	0.06	12	2	0.20 (0.02–0.89)	0.02	15	3	0.27 (0.05–0.97)	0.02	15	3	0.33 (0.06–1.17)	0.05	17	3	0.33 (0.06–1.13)	0.04
Tis to T1	0	0	‐	‐	2	0	‐	‐	3	0	‐	‐	3	0	‐	‐	3	0	‐	‐
Ta to MIBC	0	0	‐	‐	2	0	‐	‐	2	2	1.37 (0.10–18.9)	0.56	2	2	1.65 (0.11–22.8)	0.48	2	2	1.85 (0.13–25.5)	0.44
Tis to MIBC	0	0	‐	‐	1	0	‐	‐	1	0	‐	‐	1	0	‐	‐	1	0	‐	‐
T1 to MIBC	1	1	1.06 (0.01–83.1)	0.74	3	3	1.18 (0.16–8.8)	0.57	4	3	1.03 (0.15–6.1)	0.63	4	3	1.24 (0.18–7.33)	0.53	4	3	1.39 (0.20–8.20)	0.47
Grade‐up																				
LG to HG (including CIS)	6	4	0.71 (0.15–3.0)	0.41	12	7	0.69 (0.23–1.9)	0.29	15	7	0.64 (0.22–1.67)	0.22	16	7	0.72 (0.25–1.86)	0.31	21	7	0.62 (0.22–1.50)	0.18

Abbreviations: CI, confidence interval; CIS, carcinoma in situ; LG, low grade; HG, high grade; MIBC, muscle‐invasive bladder cancer; TURBT, transurethral resection of the bladder tumour; OR, odds ratio; PDD, photodynamic diagnosis; PFS, progression‐free survival; WL, white‐light.

**FIGURE 3:**
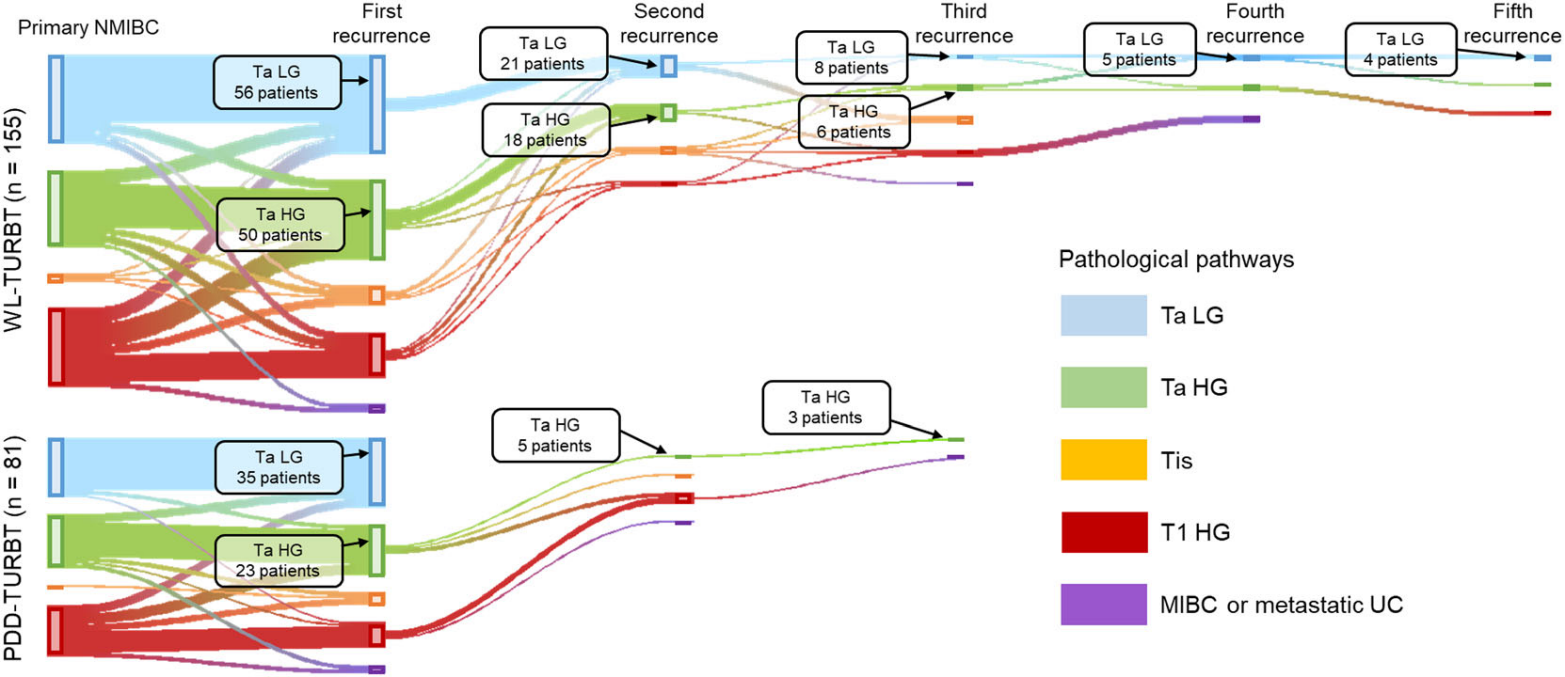
Sankey diagrams connecting the initial diagnosis of NMIBC and subsequent pathological pathways according to the use of photodynamic diagnosis. Of 990 patients (495 per group), 155 in the WL‐PDD‐TURBT group (upper panel) and 81 in the PDD‐TURBT group (lower panel) had at least one instance of bladder recurrence and/or progression. Each vertical bar represents a pathological category: Ta low‐grade (LG), Ta high‐grade (HG), isolated Tis, and T1 HG. The height of the bars is proportional to the number of patients. TURBT, transurethral resection of bladder tumour; WL, conventional white‐light; PDD, photodynamic diagnosis‐assisted; NMIBC, non‐muscle‐invasive bladder cancer; UC, urothelial carcinoma

For the 236 patients who developed bladder recurrence and/or progression at least once, we connected the initial NMIBC diagnosis and subsequent pathological pathways in Sankey diagrams (Figures 3 and S2). This showed that patients with neither primary TaLG nor recurrent TaLG had direct c‐progression. The Sankey diagrams also indicated that IBCG‐progression was an important stepping‐stone to c‐progression and that most patients with c‐progression had previously had T1HG tumours. When we compared the treatment groups, we found that, in the PDD‐TURBT group, patients with primary TaLG and first‐recurrence TaLG did not have subsequent bladder recurrence or progression, whereas some of those in the WL‐TURBT group developed recurrence after treatment.

**FIGURE 4:**
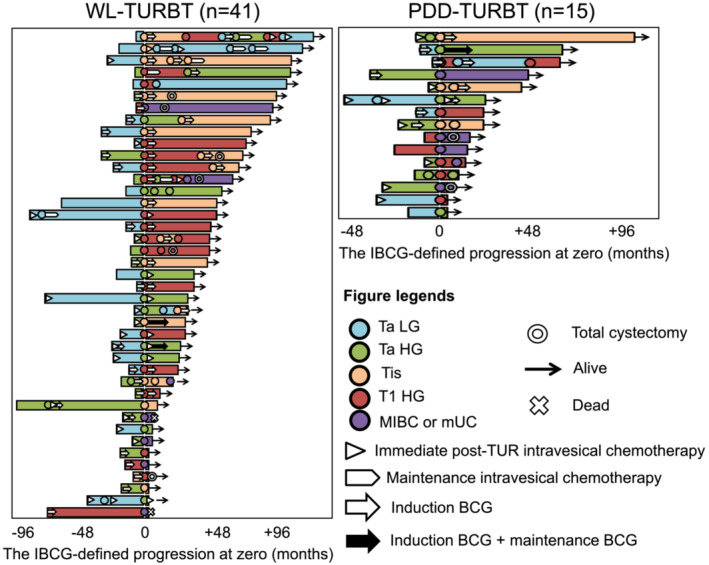
Swimmer plots of patients with IBCG‐defined progression. Of 990 patients (495 per group), 41 in the WL‐TURBT group (left panel) and 15 in the PDD‐TURBT group (right panel) demonstrated IBCG‐defined progression. Swimmer plots before and after the onset of IBCG‐defined progression indicate the adjuvant treatments, treatment‐free duration, sequential treatments, cystectomy‐free duration, and overall survival. TURBT, transurethral resection of bladder tumour; WL, conventional white‐light; PDD, photodynamic diagnosis‐assisted; MIBC, muscle‐invasive bladder cancer; UC, urothelial carcinoma; BCG, bacillus Calmette‐Guerin

Lastly, we generated swimmer plots showing treatment sequences, pathway to progression, survival, and death on follow‐up for patients who had IBCG‐progression (41 and 15 patients in the WL‐TURBT and PDD‐TURBT groups, respectively; Figure 4). In the WL‐TURBT group, five patients had c‐progression at the first bladder recurrence, while two patients had repeated recurrence prior to c‐progression. In the PDD‐TURBT group, four patients had c‐progression at the first recurrence, while one patient had c‐progression after T1HG recurrence. Of note, 16/495 patients in the WL‐TURBT group (3.2%), but only five patients (1.0%) in the PDD‐TURBT group, showed progression from TaLG or TaHG to T1HG tumours.

## 4 DISCUSSION

In this study, we evaluated the real benefit of blue‐light PDD in TURBT for NMIBC, based on progression defined using the IBCG criteria, and investigated the subsequent pathways to progression in patients who underwent WL‐TURBT or PDD‐TURBT. Based on comparison of propensity score‐matched groups, we showed that PDD use reduced the BC recurrence as well as the IBCG‐progression risk, but not c‐progression. This was apparently due to a reduction in stage‐up from Ta to T1 and in grade‐up from LG to HG tumours. Sankey diagrams showed that recurrence or progression did not occur in patients with primary or first recurrence of TaLG tumours in the PDD‐TURBT group, whereas some patients in the WL‐TURBT group developed recurrence post‐treatment.

The beneficial effect of PDD‐TURBT on the BC recurrence rate in NMIBC patients has been proven in several randomized control trials (RCTs), retrospective studies, and systematic reviews and meta‐analyses.^4^, ^5^, ^6^, ^7^, ^11^, ^12^, ^20^, ^21^, ^22^ In contrast, evidence regarding the positive effect of this treatment on c‐progression and related mortality is limited. One RCT demonstrated a reduction in both bladder recurrence and c‐progression risk with PDD‐TURBT as compared to with WL‐TURBT (HR 0.56, 95% CI 0.39–0.80, *P* = 0.001 and HR 0.33, 95% CI 0.12–0.91, *P* = 0.031, respectively), whereas the OS and cancer‐specific survival rates remained similar.^23^ A network meta‐analysis for progression rates found that neither ALA‐based PDD‐TURBT (odds ratio 0.97, 95% CI 0.57–1.62), nor HAL‐based PDD‐TURBT (odds ratio 0.71, 95% CI 0.37–1.47) could reduce the progression risk as compared to WL‐TURBT.^6^

In major prospective clinical trials and retrospective studies, the most commonly used definition of NMIBC progression has been the development of MIBC.^8^ The IBCG pointed out that this conventionally used definition failed to include other clinically important aspects of cancer advances, including progression to lamina propria invasion (Ta or Tis to T1) and grade‐up (LG to HG). A previous study reanalysed patient data from an earlier Phase III multicentre study^22^ by using IBCG‐progression, and compared the progression rate between conventional WL‐TURBT (*n* = 261) and PDD‐TURBT (*n* = 255).^12^ The PDD‐TURBT group showed a lower rate of progression at the 4.5 years follow‐up (*P* = 0.085, vs. WL‐TURBT) and prolonged time to progression (*P* = 0.05, vs. WL‐TURBT).

Uniquely, this study evaluated nine endpoints (Figure 2). Consistent with previous evidence, we found that PDD‐TURBT provided a strong benefit in terms of reducing bladder recurrence, but not c‐progression. Recurrent TaLG tumours and any recurrent HG tumours were categorized in the low‐ or intermediate‐risk group and high‐risk group, respectively, in the clinical practice guidelines.^1^, ^2^, ^3^ Therefore, we focused on the beneficial effect of PDD‐TURBT on HG tumour recurrence, and demonstrated that PDD‐TURBT decreased the risk of HG tumour recurrence in particular, and not only that of LG tumour recurrence. In addition, IBCG‐PFS and grade‐up‐free survival was significantly longer in the PDD‐TURBT group than in the WL‐TURBT group. We compared progression rates during short‐term and long‐term follow‐up, according to progression patterns (Table 1). In the 2‐year follow‐up analysis, PDD use was associated with decreased risk of the IBCG‐progression (33 in WL‐TURBT vs. 12 in PDD‐TURBT, respectively). This finding was apparently attributed to a decreased risk of stage‐up, from Ta to T1 (12 vs. 2), and of grade‐up, from LG to HG (12 vs. 7). A similar finding was noted in the 3‐, 5‐, and 10‐year follow‐up analyses.

A previous RCT conducted by Geavlete et al. concluded that BC recurrence rates during short‐term follow‐up (at 3 months, and 1 and 2 years) were significantly improved with the HAL‐based PDD‐TURBT.^24^ They emphasized that the additional lesions identified with the assistance of PDD significantly modified the recurrence‐ and progression‐risk categories as well as the subsequent treatment in a substantial percentage of patients (19%). Selecting patients who are likely to benefit from intravesical BCG therapy or chemotherapy and who require postoperative treatment is essential in the management of NMIBC. The PDD‐TURBT group in our cohort may include patients who received appropriate guideline‐recommended postoperative treatment because of additionally found lesions, which resulted in better clinical outcomes.

We generated some Sankey diagrams to visualize the pathological pathways of progression from primary NMIBC and investigated possible differences in these pathways between the WL‐TURBT and PDD‐TURBT groups (Figure 3). This tool clearly demonstrated that TaLG tumours did not recur after first recurrence in the PDD‐TURBT group, while repeated recurrence of TaLG was detected in the WL‐TURBT group. We and another research group reported that PDD assistance reduced not only the rate of the first bladder recurrence but also that of subsequent repeated bladder recurrence. In our two previous studies,^11^, ^20^ the person‐time method with 10 000 person‐follow‐up days revealed a 63% reduction of recurrence, from 10.5 (WL‐TURBT) to 3.9 (PDD‐TURBT) and a 65% reduction, from 12.8 to 5.8. Similar result was observed in this large‐scale study (Table 1). Highly‐sensitive PDD detection of tiny lesions, which are frequently overlooked by conventional WL observation, can improve the rate of complete resection, reducing the risk of repeated bladder recurrence, particularly for TaLG tumours. Considering the psychological and physical stress on patients and the medical cost involved, more effort should be made to research the natural history of repeated recurrence of bladder after initial TURBT and to decrease the risk of repeated recurrence.

This study had several limitations. Its retrospective nature implied an inherent potential for selection bias; for example, the treatment choice and follow‐up protocol depended on the institution and the physician's discretion. The cohort was derived from multiple academic hospitals, which might introduce inconsistencies in surgical skills, clinical interpretations, and pathological diagnoses. We enrolled patients who were treated between 2006 and 2020. During this decade, the treatment strategies, modalities, and surgical skills changed over time, which could influence the outcomes.

To the best of our knowledge, no previous study has described real‐world evidence regarding the potential benefit of PDD assistance in NMIBC progression defined by IBCG criteria. The progression rate at 1 year after treatment was significantly decreased in the PDD‐TURBT group as compared to the WL‐TURBT group. In addition, using Sankey diagrams, we investigated the difference in pathological pathways after initial TURBT between the WL‐TURBT and PDD‐TURBT groups, and demonstrated that repeated recurrence could be prevented by using PDD. Further large‐scale studies are required to understand the real clinical value of PDD‐TURBT better.

## CONFLICT OF INTEREST

The authors disclose no potential conflicts of interest.

## AUTHOR CONTRIBUTIONS

MM, the first author, made significant contributions to the research design, data collection and interpretation, and writing of the original manuscript. NN and TN performed the formal analysis. KN, MO, YM, HM, HF, KI, KK, HirM, and TF performed the data curation. HidM, YH, and KF were involved in interpretation of the data and contributed important intellectual input in manuscript writing—review and editing. All of the authors meet criteria for authorship, have read the manuscript, and have approved this submission.

## Supporting information


**Figure S1:** Comparison of survival curves between the unadjusted WL‐TURBT (n = 1057) and PDD‐TURBT (n = 497) groups.
Survival curves for the nine oncological outcomes were generated from the date of initial TURBT (diagnosis of bladder cancer). Survival rates were estimated using the Kaplan–Meier method and hazard ratios (HRs) with 95% confidence intervals (CIs) were calculated using the log‐rank test.
TURBT, transurethral resection of bladder tumour; WL, conventional white‐light; PDD, photodynamic diagnosis‐assisted.

**Figure S2:** Sankey diagrams connecting the initial diagnosis of NMIBC and subsequent pathological pathways.
Of 990 patients, 236 had at least one instance of bladder cancer recurrence and/or progression. Each vertical bar represents a pathological category: Ta low‐grade (LG), Ta high‐grade (HG), isolated Tis, and T1HG. The height of the bars is proportional to the number of patients.
TURBT, transurethral resection of bladder tumour; WL, conventional white‐light; PDD, photodynamic diagnosis‐assisted; NMIBC, non‐muscle‐invasive bladder cancer; UC, urothelial carcinoma.

**Table S1.** Characteristics of study patients and a comparison between the WL‐TURBT group and PDD‐TURBT group: before and after propensity‐score matching adjustment.

## Data Availability

The datasets used and/or analysed during the present study are available from the corresponding author on reasonable request.
