# Supplementary material for: Additional oncological benefit of photodynamic diagnosis with blue light cystoscopy in transurethral resection for primary non‐muscle‐invasive bladder cancer: A comparative study from experienced institutes
Source: BJUI Compass. 2023 Jan 13;4(3):305–13. doi: 10.1002/bco2.215 (PMC10071077; doi:10.1002/bco2.215)
Supplement: Supplementary file 1 — Figure S1: Comparison of survival curves between the unadjusted WL‐TURBT (n = 1057) and PDD‐TURBT (n = 497) groups. Survival curves for the nine oncological outcomes were generated from the date of initial TURBT (diagnosis of bladder cancer). Survival rates were estimated using the Kaplan–Meier method and hazard ratios (HRs) with 95% confidence intervals (CIs) were calculated using the log‐rank test. TURBT, transurethral resection of bladder tumour; WL, conventional white‐light; PDD, photodynamic diagnosis‐assisted. [file BCO2-4-305-s002.pptx]

## Slide 1
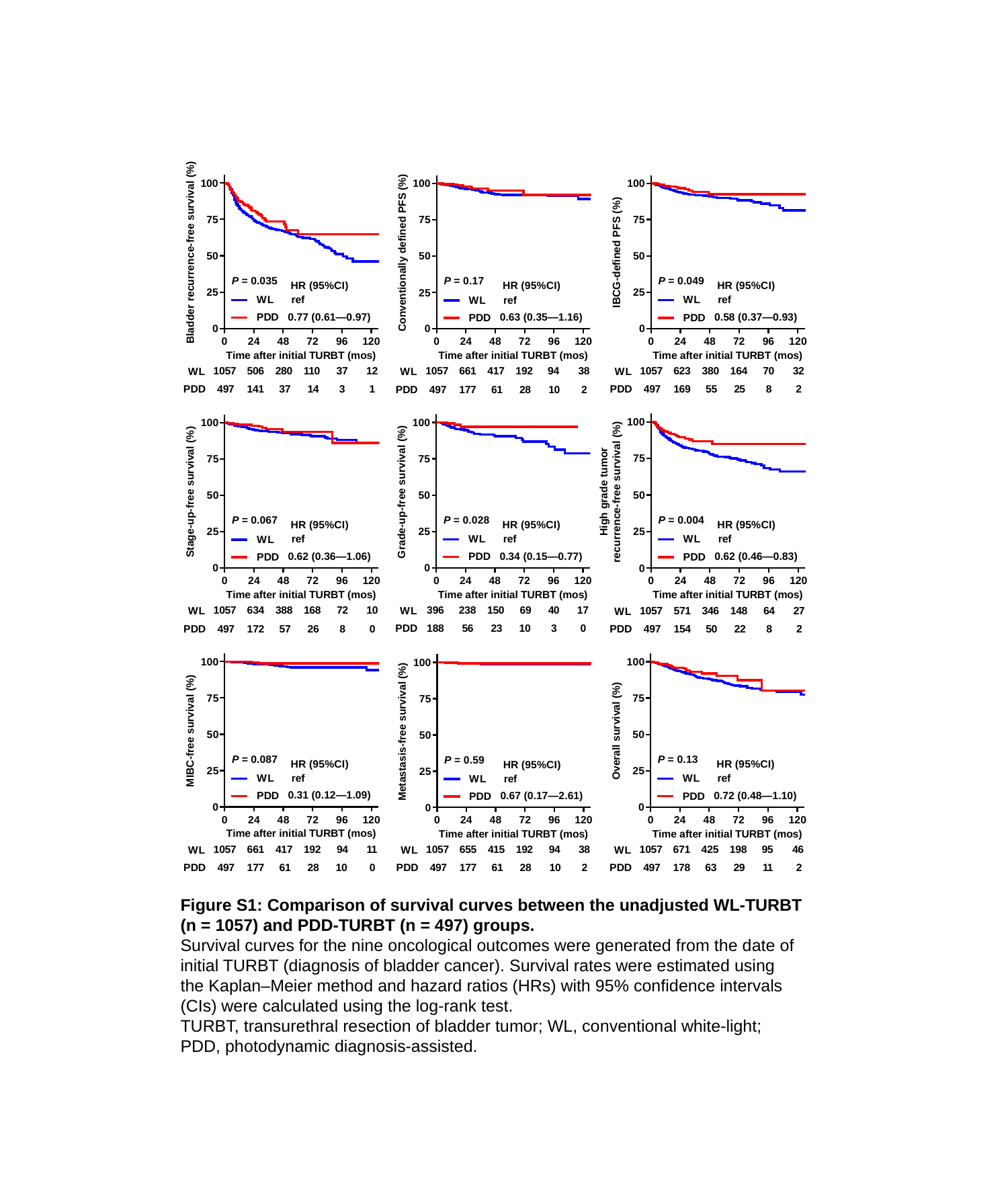

Figure S1: Comparison of survival curves between the unadjusted WL-TURBT (n = 1057) and PDD-TURBT (n = 497) groups.
Survival curves for the nine oncological outcomes were generated from the date of initial TURBT (diagnosis of bladder cancer). Survival rates were estimated using the Kaplan–Meier method and hazard ratios (HRs) with 95% confidence intervals (CIs) were calculated using the log-rank test.
TURBT, transurethral resection of bladder tumor; WL, conventional white-light; PDD, photodynamic diagnosis-assisted.
