# Supplementary material for: Additional oncological benefit of photodynamic diagnosis with blue light cystoscopy in transurethral resection for primary non‐muscle‐invasive bladder cancer: A comparative study from experienced institutes
Source: BJUI Compass. 2023 Jan 13;4(3):305–13. doi: 10.1002/bco2.215 (PMC10071077; doi:10.1002/bco2.215)
Supplement: Supplementary file 2 — Figure S2: Sankey diagrams connecting the initial diagnosis of NMIBC and subsequent pathological pathways. Of 990 patients, 236 had at least one instance of bladder cancer recurrence and/or progression. Each vertical bar represents a pathological category: Ta low‐grade (LG), Ta high‐grade (HG), isolated Tis, and T1HG. The height of the bars is proportional to the number of patients. TURBT, transurethral resection of bladder tumour; WL, conventional white‐light; PDD, photodynamic diagnosis‐assisted; NMIBC, non‐muscle‐invasive bladder cancer; UC, urothelial carcinoma. [file BCO2-4-305-s003.pptx]

## Slide 1
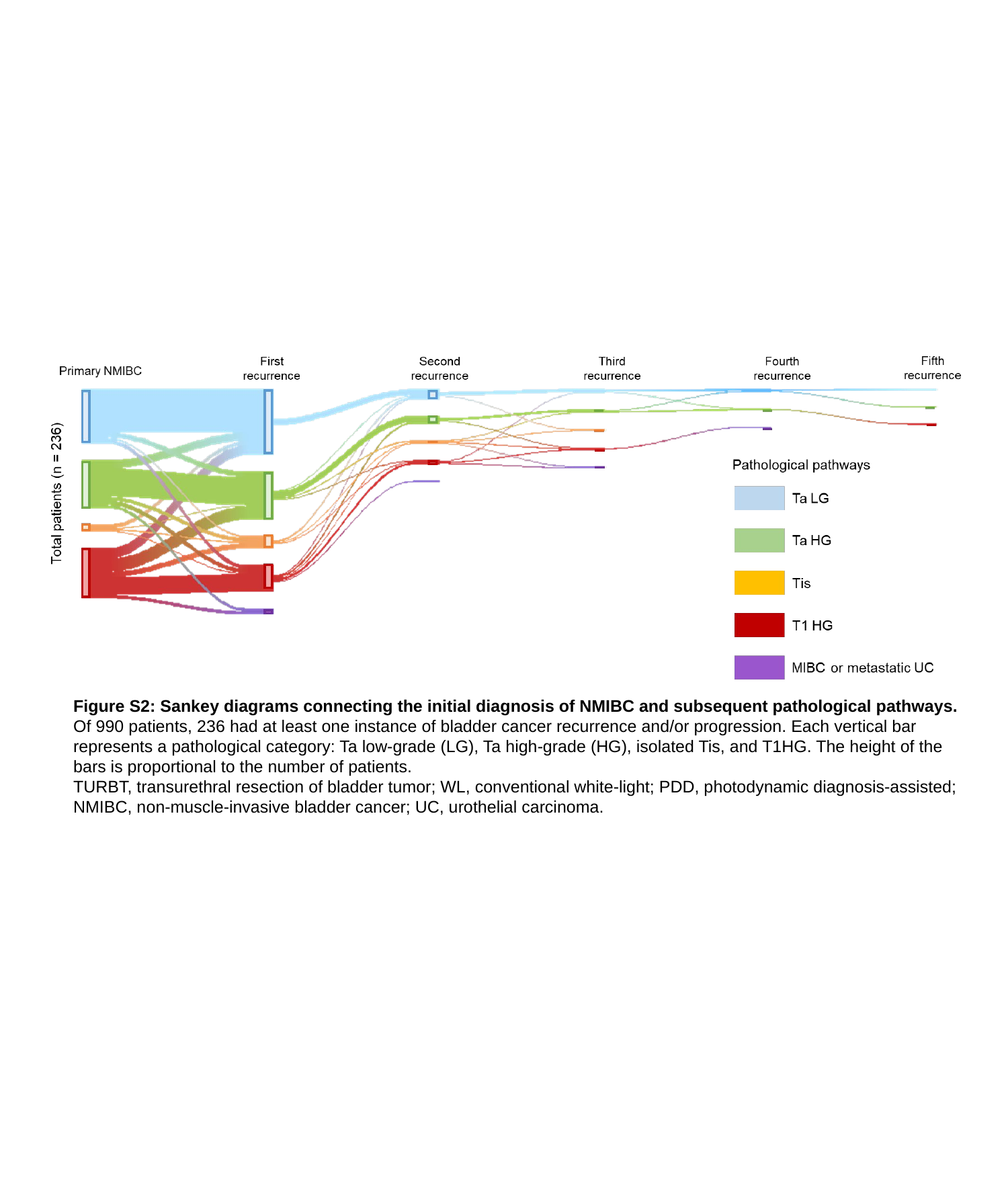

Figure S2: Sankey diagrams connecting the initial diagnosis of NMIBC and subsequent pathological pathways.
Of 990 patients, 236 had at least one instance of bladder cancer recurrence and/or progression. Each vertical bar represents a pathological category: Ta low-grade (LG), Ta high-grade (HG), isolated Tis, and T1HG. The height of the bars is proportional to the number of patients.
TURBT, transurethral resection of bladder tumor; WL, conventional white-light; PDD, photodynamic diagnosis-assisted; NMIBC, non-muscle-invasive bladder cancer; UC, urothelial carcinoma.
