# Supplementary figures and images for: Additional oncological benefit of photodynamic diagnosis with blue light cystoscopy in transurethral resection for primary non‐muscle‐invasive bladder cancer: A comparative study from experienced institutes
Source: BJUI Compass. 2023 Jan 13;4(3):305–13. doi: 10.1002/bco2.215 (PMC10071077; doi:10.1002/bco2.215)

## Slide 1
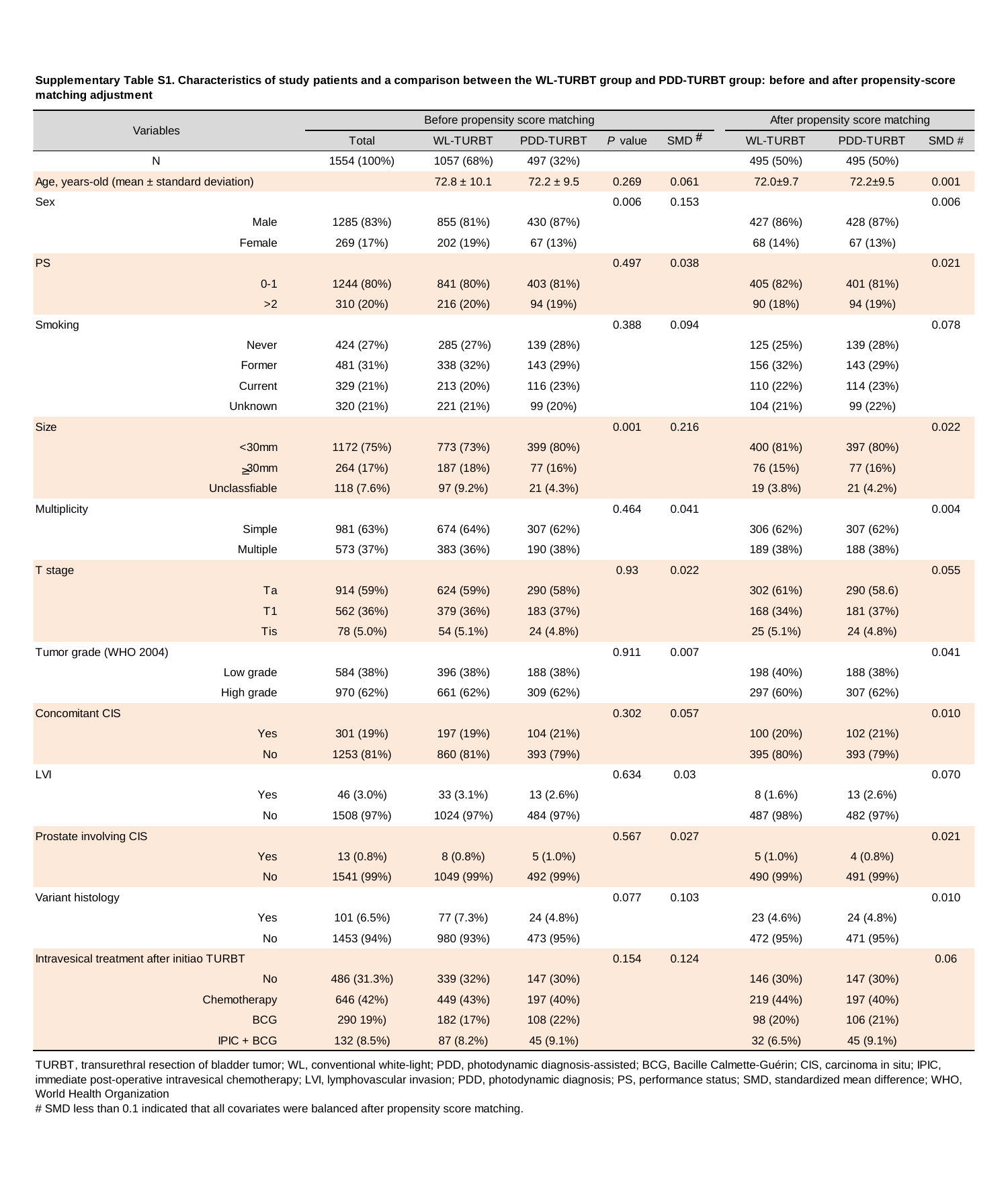

Supplement: Supplementary file 3 — Table S1. Characteristics of study patients and a comparison between the WL‐TURBT group and PDD‐TURBT group: before and after propensity‐score matching adjustment. [file BCO2-4-305-s001.pptx]
